# What can we offer to 3 million MDRTB household contacts in 2016?

**DOI:** 10.1186/s12916-016-0610-x

**Published:** 2016-04-01

**Authors:** David A. J. Moore

**Affiliations:** TB Centre, London School of Hygiene and Tropical Medicine, Keppel Street, London, WC1E 7HT UK

**Keywords:** Multidrug resistant tuberculosis, Latent tuberculosis infection, Household contacts

## Abstract

The diagnosis of multidrug resistant tuberculosis (MDR-TB) in any individual is the beginning of a prolonged and difficult therapeutic journey. It also marks the moment from which to begin consideration of how to manage close contacts. Preventive therapy for drug-susceptible latent tuberculosis infection has been demonstrated to be effective at reducing the risk of future disease; the stakes are higher when considering prevention of MDR-TB because treatment of active disease is more prolonged and toxic and much less effective. This has encouraged exploration of the potential utility of preventive therapy, with second-line agents, in reducing future incident drug-resistant TB.

Three clinical trials of preventive therapy for contacts of patients with MDR-TB are starting in 2015/16; results will not be available until at least 2020, so what should be offered to exposed contacts in the interim?

A recent policy brief, arising from a global consultation meeting of international experts, recommended preventive therapy based upon very limited available observational data. However the many known unknowns associated with this approach, include the high proportion of index-contact pairs with discordant drug susceptibility profiles and (even if susceptibilities are shared) the lack of data supporting the use of the selected agents in the treatment of latent infection (rather than active disease).

It is important to acknowledge that the alternative to offering preventive therapy is not doing nothing. On the contrary, identified contacts should be maintained under close, active surveillance for 24 months, enabling early detection of active disease in the small proportion amongst whom this may occur. Such patients should benefit from less extensive disease at diagnosis and early access to individualized therapeutic regimens with improved treatment outcomes. Moreover the vast majority of contacts that do not develop disease will benefit from avoidance of potentially toxic, unnecessary therapy.

Whether preventive therapy or close observation are implemented, national programmes should maintain a register of all contacts, interventions and 24 month outcomes; these will provide important performance metrics for programmatic management of MDRTB. If harmonized and standardized internationally, such a register could rapidly yield a wealth of observational data, to complement the trial results of the future.

## MDRTB in 2015

Multidrug resistant tuberculosis (MDRTB) is defined as TB resistant to isoniazid and rifampicin. The World Health Organisation estimated that there were around 480,000 incident cases of MDRTB in 2014 [1]. In contrast to TB susceptible to these agents, which is effectively treated with a six-month course of generally well tolerated chemotherapy, current recommendations for MDRTB require treatment for a minimum of 20 months with a combination that includes a daily injectable agent for at least the first eight months [2]. Such regimens are highly toxic and poorly tolerated with the additional sting of poor efficacy. Globally only 50 % of MDRTB patients achieve a successful treatment outcome (cure or completion of treatment) [1]. Many of the 190,000 people estimated to have succumbed to MDRTB in 2014 died even before a diagnosis could be made. There is much work to be done to increase MDRTB detection, to enhance linkage of diagnosis to initiation of appropriate treatment, and to improve efficacy and tolerability of MDRTB therapy. However there is cause for optimism. Scale-up of diagnostics for MDRTB continues, even if the goal of universal drug susceptibility testing (DST) still seems a distant, unaffordable aspiration, and new regimens [3] and agents [4, 5] for MDRTB therapy hold the promise that shorter, exclusively oral regimens may become the standard of care within a few years.

## The elephant in the room

That many more patients are being diagnosed with MDRTB each year is a good thing, representing much improved case ascertainment rather than necessarily a growing problem (though both may be true). Patients are more likely to receive appropriate therapy, reducing morbidity and mortality at the individual level and interrupting transmission earlier at the population level. However there is a responsibility that accompanies every MDRTB diagnosis that is currently being shirked by most national TB control programmes (NTPs), overwhelmed as they often are by competing priorities, and that is how to manage MDRTB-exposed household contacts.

Here, we are in a data desert, and guidance on prevention of MDRTB from the World Health Organisation is not particularly illuminating (“(1) Early detection and high quality treatment of drug susceptible TB, (2) early detection and high quality treatment of drug resistant TB, (3) effective implementation of infection control measures, (4) strengthening and regulation of health systems, (5) addressing underlying risk factors and social determinants” [2]).

As a minimum all household contacts should be identified and screened for active TB disease soon after index case diagnosis, and rapid drug susceptibility testing should be performed for all co-incident cases. The high yield of active case finding within TB-affected households [6, 7] is particularly valuable if additional drug-resistant cases can be removed from the pool of infectious individuals sustaining transmission within the community. After identification of co-incident household cases the next step is to decide what, if anything, can be offered to the remaining MDR-exposed household contacts to manage their risk of future MDRTB.

## What are the options?

### Lessons from management of drug-susceptible latent infection

It is worth considering what can be learnt from the approach taken when the index case has drug susceptible disease. For close contacts of index cases known to have drug-susceptible TB (susceptible to rifampicin and isoniazid) who are deemed to be at high risk of progression to active disease – young children, HIV-infected, immunosuppressed – preventive therapy with isoniazid is recommended [8, 9]. There is strong evidence that this affords good protection against subsequent TB disease in well-defined populations [10]. In low incidence settings where the force of infection is low this is presumed to be mediated through sterilization of existing latent infection preventing reactivation disease, whereas in high burden settings waning efficacy after PT is discontinued [11] suggests that there may also be an effect mediated whilst taking therapy through prevention of the establishment of further infection on re-exposure [12].

Most studies demonstrate that PT efficacy is greatest amongst subjects with a positive tuberculin skin test (TST) [13], though this is not a universal finding [14], Moreover the discourse about TST-negative subjects, particularly amongst patients with HIV, fails to adequately recognise that this group is a mix of those who can correctly be assumed to not have LTBI and those unable to mount a response to any antigen, not just tuberculin, by virtue of profound immunodeficiency and who may therefore be at particularly high risk of reactivation of unrecognised LTBI. Neither TST nor interferon-gamma release assays (IGRAs) serve us well in deciding who is at highest risk of future progression to active TB. Moreover the need for such a gateway test has been a major impediment to the scale-up of isoniazid preventive therapy [15] as part of the World Health organisations “3 I s” for tackling HIV-associated TB. All of which is to say that in consideration of how to manage MDRTB household contacts, for whom the stakes may be somewhat higher, it should not be assumed that decision making necessarily starts with (flawed) testing for LTBI.

### What then can be offered to MDRTB contacts?

An ideal preventive therapy regimen should include a second line agent(s) which is (1) effective against non- or slowly replicating organisms and (2) for which there is a high likelihood that the infecting strain is susceptible. On the first point it is noteworthy that had the mechanism of action of isoniazid [16], a drug which only kills actively growing mycobacteria, been understood at the time it is doubtful it would ever have been proposed for preventive therapy. Arguably therefore, whilst intuitively desirable, this may not be an absolute requirement.

For the second point it comes down to probability, because there is currently no way to determine the drug susceptibility of the LTBI strain. What is the likelihood that the strain of an MDR contact with LTBI will have a drug susceptibility test (DST) profile that is no more resistant than that of the index case, and therefore potentially treatable with an agent(s) selected on the basis of index case DST? The only data we have is from studies of second cases amongst household contacts. Whilst it might be argued that these cases may not be representative of all MDR LTBI – drug resistance conferring mutations exacting a “fitness cost” on some infecting strains [17, 18], reducing their capacity for reactivation, and thus remaining invisible – the strains of those second cases are nevertheless precisely those that should be targeted.

Studies examining DST concordance (genotypic or phenotypic) between index MDRTB and subsequent incident TB cases in the same household have been largely conducted in high TB burden settings where the risk of infection outside the household is also important. The proportion of within-household second (rather than secondary) cases that do not share the index case DST varies widely (in studies with >10 DST pairs tested, concordance was 18, 60, 62, 72, 88 % [19]), indicating infection was acquired elsewhere. Concordance is higher amongst closer contacts who share the same bedroom and contacts with HIV or diabetes and when the index case is smear-positive [7] but beyond this there is little data on characteristics of index case, contact, strain or household environment that help to estimate the likelihood that a contact will share the same strain.

Given the significantly lower efficacy and greater toxicity of second line agents used in the treatment of MDRTB it is essential that immediate DST be performed for all second cases to ensure the correct regimen is initiated. The implication for preventive therapy decision-making is also clear: it is incorrect to assume that a second line agent would be necessary for all MDRTB contacts with LTBI needing preventive therapy. However, conversely whilst isoniazid would not be expected to be effective in MDRTB LTBI, use of a second line agent effective against latent MDRTB strains should equally effectively sterilize non-MDR strains. Thus if the selected second line agent were well tolerated, the index-contact question becomes less about concordance and more about the much lower probability that the contact case harbours an LTBI strain which is more resistant than the index case. Tailored preventive therapy, selecting a drug by estimating the probability of strain susceptibility would pose a major operational challenge and since data on therapeutic efficacy will only be available for a few agents within the next 5-10 years such an approach would probably be better used to determine whether success is likely for the few drugs available.

### What candidate agents may be suitable?

Several clinical trials of preventive therapy have been scheduled to commence in 2015 and 2016 (Table 1); protocol regimens and study populations have understandably evolved as observational evidence has grown and new agents have become available. Levofloxacin is the current poster-boy – available in a generic formulation, once daily dosing and well tolerated by all age groups [20], with good anti-mycobacterial activity against susceptible strains. That one fifth of MDR strains tested in 2014 demonstrated quinolone resistance [1] (albeit only representing one quarter of globally notified cases) raises cause for concern about the long-term viability of use of this class of drug, which is widely available over the counter in many high TB burden countries. Interest in incorporating delamanid, a new nitroimidazole [5], and one of two novel anti-tuberculosis agents approved in recent years, into the planned trials, highlights the reservations of investigators about both the long term durability of quinolone utility and biological plausibility about activity against non-replicating organisms of agents that operate through interference with DNA gyrase [21].

**Table 1:** Randomised controlled trials of preventive therapy for MDRTB household contacts (HIV-infected or HIV-uninfected) expected to commence recruitment in 2015

Name of trial	Location	Population	Intervention and comparator	Months of treatment/follow-up	Clinical trials registry #
TB CHAMP	South Africa	Children <5 years	Levofloxacin vs. placebo	6/18	-
PHOENix	ACTG sites	All contacts (Adults and children)	Delamanid vs. isoniazid	6/22	-
V-QUIN	Vietnam	All contacts (Adults and children)	Levofloxacin vs. placebo	6/30	ACTRN12616000215426

Disappointingly there does not seem to be a current appetite for a bolder, short duration option, such as has recently been demonstrated for drug-susceptible LTBI in which 12 doses of rifapentine plus isoniazid administered once weekly over 3 months performs as well as 270 doses of daily isoniazid over 9 months [22].

However these trials ultimately finalise their intervention designs it is to be hoped that there will be an attempt at this early stage to harmonise subgroup eligibility criteria and collection of core treatment outcome data so that on completion all may contribute to a coherent body of evidence. Questions around operational feasibility, such as whether direct observation of therapy is needed or how to respond if quinolone resistance is subsequently identified in the index case, will also be key to understanding how to translate the research findings into future policy and practice.

### Trials will not start reporting until at least 2020 so how should MDRTB contacts be managed now?

This question was central to a meeting held in Dubai in April 2015 (“Global Consultation on Best Practices in the Delivery of Preventive Therapy for Households Exposed to Drug-Resistant Tuberculosis”), from which a policy brief was published in November 2015 [23]. Following review and discussion of published and unpublished data a set of seven guiding principles was laid out: (1) define common terms, (2) identify all household contacts, (3) evaluate all exposed individuals for TB disease, (4) offer treatment for MDRTB infection, (5) follow all exposed individuals for at least 18 months, (6) build a programmatic strategy to treat MDRTB infection, (7) learn from the experiences in treating DS-TB infection. The treatment recommended, based upon the majority opinion of the gathered experts, was a fluoroquinolone for at least 6 months either alone or in combination with ethambutol or ethionamide (according to index case DST profile). In total the observational data reports upon 650 patients treated with a wide variety of regimens, at least one third of whom received three agents for six months and another third who received pyrazinamide-containing regimens with high toxicity-related discontinuation (thus pyrazinamide is not recommended). The remaining 256 preventive therapy treated patients thus represent the global experience upon which the drug treatment recommendations are made, on data from observational, often retrospective studies, without adequate comparator control groups.

### What alternatives to preventive therapy can be offered?

The presentation of recommendations [23] in the high-quality-evidence vacuum is to be welcomed, particularly when they are the considerations of a group representing most of the global experience and interest in addressing this issue. However the alternative to preventive therapy, on which considerable emphasis is placed in the policy brief, is not to do nothing at all. On the contrary, every identified contact should be registered and actively followed up for 2 years (the period of maximum risk) to enable early detection of active TB disease and rapid initiation of appropriate DST-directed therapy.

Previously, the typical patient with MDRTB will have endured months to years of ineffective treatment with a clinical presentation characterised by significant systemic illness (cachexia, anaemia), extensive lung damage with sanctuary sites hard to reach with drug therapy, and high mycobacterial bacillary burden. The adage that prevention is better than cure would clearly apply in this situation – even a modestly effective preventive regimen that could avert the drug toxicity, patient morbidity and mortality, community-level transmission and health system cost would be worthwhile.

However the growing availability of DST should drive the “typical” patient towards a less advanced disease phenotype at the onset of MDRTB therapy, which intuitively should translate into higher rates of treatment success (even if data demonstrating this effect are so far lacking (Harris RC, Allen V, Miller AJP, et al: A systematic review of the effect of early versus late treatment initiation on the outcomes of patients treated for multidrug resistant tuberculosis, submitted)). Moreover patients identified within a few months of symptom onset by virtue of close NTP surveillance may respond very well to MDRTB treatment (with shorter duration of infectiousness), including with the shorter regimens. This, and the recognition that 90 % of contacts will never progress to active disease and thus would receive preventive therapy unnecessarily, shifts the risk-benefit balance back towards close contact surveillance rather than preventive therapy of unknown efficacy. This also plays into the question of which contacts should be offered preventive therapy – people living with HIV, young children, all contacts? The number needed to treat to prevent a single case rapidly increases in the absence of risk factors for progression.

### Conclusions - what should national TB control programmes be doing now?

For now, early diagnosis and treatment remains the key to minimizing MDRTB morbidity and transmission. In this regard the high yield of co-prevalent active disease in household contacts and the high incidence of active disease in the two years following index case diagnosis are low-hanging fruit [19].

All household contacts should be identified and entered into a national registry as an integral element of the management of each patient treated for MDRTB. Future MDRTB management might better be conceived as household-centred rather than patient-centred care. The level of sophistication required to deliver MDRTB treatment includes IT capability into which an electronic registry could readily be incorporated. Ideally an internationally agreed minimum dataset for such a registry should be established and, with appropriate data protection safeguards, a curated internationally coordinated database could be developed and offered to countries.

All contacts should then be followed-up for two years from the date of index case diagnosis, whether offered and/or receiving preventive therapy or not. The form of follow up could include symptom screening, sputum culture or Xpert MTB/RIF or radiology as decided locally according to resource availability; contact frequency, even if only for a quick symptom screen, should be at least quarterly to maximise the chance of early diagnosis. All investigations, diagnoses, therapies and outcomes could be readily collected into the registry, which would rapidly become both an auditable resource generating metrics by which NTPs could evaluate their programmatic management of drug resistant tuberculosis (PMDT) and a valuable source of detailed, systematically collected, unbiased observational data. At an international level every MDRTB contact would thus contribute data to a rapidly enlarging knowledge and evidence-base. Whilst awaiting the high quality evidence from randomised trials we could at least reassure ourselves that we were doing the very best that we could for the 3 million-plus household contacts around the globe who are asking “what about us?”.

### Ethics approval and consent to participate

Not applicable.

### Consent for publication

Not applicable.

### Availability of data and materials

Not applicable, no original data.

## Abbreviations

DST: drug susceptibility testing
DS-TB: drug susceptible tuberculosis
IGRA: interferon gamma release assay
LTBI: latent tuberculosis infection
MDRTB: multidrug resistant tuberculosis
NTP: national tuberculosis control programme
TST: tuberculin skin test
